# Comparison of Heterologous Prime-Boost Strategies against Human Immunodeficiency Virus Type 1 Gag Using Negative Stranded RNA Viruses

**DOI:** 10.1371/journal.pone.0067123

**Published:** 2013-06-26

**Authors:** Tessa M. Lawrence, Celestine N. Wanjalla, Emily A. Gomme, Christoph Wirblich, Anthony Gatt, Elena Carnero, Adolfo García-Sastre, Douglas S. Lyles, James P. McGettigan, Matthias J. Schnell

**Affiliations:** 1 Department of Microbiology and Immunology, Jefferson Medical College, Thomas Jefferson University, Philadelphia, Pennsylvania, United States of America; 2 Department of Microbiology, Icahn School of Medicine at Mount Sinai, New York, New York, United States of America; 3 Global Health and Emerging Pathogens Institute, Icahn School of Medicine at Mount Sinai, New York, New York, United States of America; 4 Department of Medicine, Icahn School of Medicine at Mount Sinai, New York, New York, United States of America; 5 Department of Biochemistry, Wake Forest School of Medicine, Winston-Salem, North Carolina, United States of America; 6 Jefferson Vaccine Center, Jefferson Medical College, Thomas Jefferson University, Philadelphia, Pennsylvania, United States of America; Virginia Polytechnic Institute and State University, United States of America

## Abstract

This study analyzed a heterologous prime-boost vaccine approach against HIV-1 using three different antigenically unrelated negative-stranded viruses (NSV) expressing HIV-1 Gag as vaccine vectors: rabies virus (RABV), vesicular stomatitis virus (VSV) and Newcastle disease virus (NDV). We hypothesized that this approach would result in more robust cellular immune responses than those achieved with the use of any of the vaccines alone in a homologous prime-boost regimen. To this end, we primed BALB/c mice with each of the NSV-based vectors. Primed mice were rested for thirty-five days after which we administered a second immunization with the same or heterologous NSV-Gag viruses. The magnitude and quality of the Gag-specific CD8^+^ T cells in response to these vectors post boost were measured. In addition, we performed challenge experiments using vaccinia virus expressing HIV-1 Gag (VV-Gag) thirty-three days after the boost inoculation. Our results showed that the choice of the vaccine used for priming was important for the detected Gag-specific CD8^+^ T cell recall responses post boost and that NDV-Gag appeared to result in a more robust recall of CD8^+^ T cell responses independent of the prime vaccine used. However, the different prime-boost strategies were not distinct for the parameters studied in the challenge experiments using VV-Gag but did indicate some benefits compared to single immunizations. Taken together, our data show that NSV vectors can individually stimulate HIV-Gag specific CD8^+^ T cells that are effectively recalled by other NSV vectors in a heterologous prime-boost approach. These results provide evidence that RABV, VSV and NDV can be used in combination to develop vaccines needing prime-boost regimens to stimulate effective immune responses.

## Introduction

Every year, 50,000 people in the United States become infected with HIV-1 [1] and one in every 20 adults live with HIV in Sub-Saharan Africa (UNAIDS report 2012). Despite increasing knowledge of HIV as a pathogen and the interaction with the immune system [2], the conundrum is creating a vaccine against a pathogen for which no adult human has been reported to have completely cleared an infection [3], [4]. The consensus is that the search for the most effective vaccine is no easy feat partly because the correlates of complete protection are unclear. However, like other clinically significant infectious diseases such as smallpox [5], the need for a vaccine against HIV is of utmost importance in the management of the global HIV epidemic.

Theoretically, a vaccine strategy most likely to stimulate an effective response against HIV would be one that can i) efficiently deliver the antigen to the host, ii) stimulate a robust humoral and cellular immune response capable of targeting the virus and virus infected cells, and iii) establish a population of memory cells that can quickly and efficiently replicate and protect a host exposed to the actual infection [6].

There has been extensive work done on vaccine development against HIV in mice and non-human primates (NHPs). Unlike other pathogens, attenuated HIV as a vaccine has not been explored, because attempts to attenuate SIV did not prevent rhesus macaques from getting an AIDS related illness [7]. Resultingly, other modes of antigen delivery have been explored. Live attenuated viral vectors have been particularly successful in the delivery of foreign antigens to the host. Distinct from other modes of vaccines such as subunit vaccines or DNA-plasmids, attenuated live viral vectors are able to induce robust cellular and humoral responses, largely due to their ability to undergo several rounds of replication *in vivo* [8]. To date, a number of different viral vectors have been extensively characterized and studied. A significant vaccine trial using human adenovirus-5 failed to result in protection of high-risk individuals from HIV infection [9]. One of the major drawbacks of this system was thought to be the presence of pre-existing antibodies against the viral vector [10], necessitating the characterization of viral vectors with potential for use in HIV vaccine development. The goal of the project presented here was to evaluate three well-characterized NSV vectors in heterologous and homologous prime/boost experiments. The study of multiple vectors in the same laboratory environment also allows for comparisons to be made between vectors while keeping other variables static.

Negative-stranded viruses (NSV) comprise four families: *Rhabdoviridae*, which includes vesicular stomatitis virus (VSV) and rabies virus (RABV); *Paramyxoviridae*, including Newcastle disease virus (NDV), human parainfluenza virus types, Sendai virus, mumps virus and measles virus, human respiratory syncytial and metapneumoviruses; *Filoviridae* including Ebola virus and Marburg virus; and Borna disease virus in the *Bornaviridae family*. NSVs have an advantage as vaccine vectors because they replicate in the cytoplasm and therefore do not have the risk of genetic recombination with host cell DNA. Of the aforementioned viruses, RABV, VSV and NDV are well-established candidates for HIV vaccine development. Schnell et al. first established a reverse genetic system in 1994 that allowed the recovery of infectious RABV from cloned cDNA [11]. This system was used to obtain other recombinant NSV including VSV [12] and NDV [13]. The genomes of these viruses can be readily manipulated to express multiple foreign genes [14], [15]. Unlike adenovirus-5, most individuals have not been previously exposed to these viruses and are thus seronegative. This factor makes them rank highly as potential viral vaccine candidates. In addition, all these viruses have been extensively studied as potential HIV/SIV vaccine vectors and shown to induce potent immune responses [15]–[19].

RABV and VSV are single-stranded negative sense RNA viruses of the family *Rhabdoviridae*. They have simple genomes that encode 5 genes – nucleoprotein (N), phosphoprotein (P), matrix protein (M), glycoprotein (G) and the large protein (L). The SAD B19 strain of RABV is an attenuated strain used for vaccine development that has been shown to be safe in mice [15], [16], [20]–[27] and NHPs [24], [26], without any vector-induced pathogenicity. RABV vaccine vectors engineered to express HIV-1 envelope and structural proteins gp160, Gag and Pol are immunogenic in mice and NHPs [15], [16], [20]–[28]. Of note, NHPs immunized with RABV expressing SIV_mac239_ Gag-Pol and SIV_mac239_ Env were protected from AIDS-like illness when challenged with the highly pathogenic SIV _mac251_ strain compared to controls [26].

Because of its neurovirulence in animal models, VSV vaccine vectors have been attenuated to make them safe vaccine vectors using several different approaches [17]. The vectors used here are based on M protein mutants of VSV [29]–[31]. Wild-type VSV-M protein is known to suppress host expression of genes needed in the innate responses (reviewed in [32]). Single-amino acid substitutions such as the M51R substitution in M protein have resulted in vectors that activate rather than suppress innate responses [29], [33], [34]. These vectors are as immunogenic as wild-type VSV but lack neurovirulence, which also improves their safety profile [30], [31].

NDV is an avian paramyxovirus that naturally infects different avian species. NDV strains are classified into three categories according to their virulence in poultry, velogenic, mesogenic and lentogenic. Lentogenic strains do not cause disease in poultry and have been widely used as live poultry vaccines against virulent (velogenic) strains of NDV. NDV undergoes only limited rounds of replication in mammals due to its inability to inhibit innate immune responses in these hosts [35]. Lentogenic strains of NDV expressing foreign antigens have been demonstrated to be immunogenic in multiple animals, including mice [13], chicken [36], [37], sheep [38] cattle [39], and NHP [40].

In this study, we explored the utility of RABV, VSV and NDV as vaccine vectors against HIV-1 Gag in a heterologous prime-boost regimen. HIV-1 Gag specific CD8^+^ T cell induction was analyzed in different prime-boost regimens. We found that all three NSVs were capable of boosting the immune responses of one another. NDV-Gag and VSV-Gag induced significantly higher responses than RABV when used as boost vaccines. There were no notable differences post-challenge with VV-Gag. In general, all three NSVs were compatible as prime-boost vaccines.

## Materials and Methods

### Mice

Six to eight week old female BALB/c mice (NIH) were maintained at the Thomas Jefferson University Animal Facilities. All animals were handled in strict accordance with good animal practice as defined by the relevant international (Association for Assessment and Accreditation of Laboratory Animal Care (AAALAC) (Accreditation Status TJU: Full)) and national regulations (TJU Animal Welfare Assurance Number: A3085-01), and all animal work was approved by the Institutional Animal Care and Use Committee (IACUC) at Thomas Jefferson University TJU. Animal use protocols are written and approved in accordance with Public Health Service Policy on Humane Care and Use of Laboratory Animals, The Guide for the Care and Use of Laboratory Animals. TJU IACUC protocol number 414 A.

### Recombinant Vaccine Vectors

**Figure 1:**
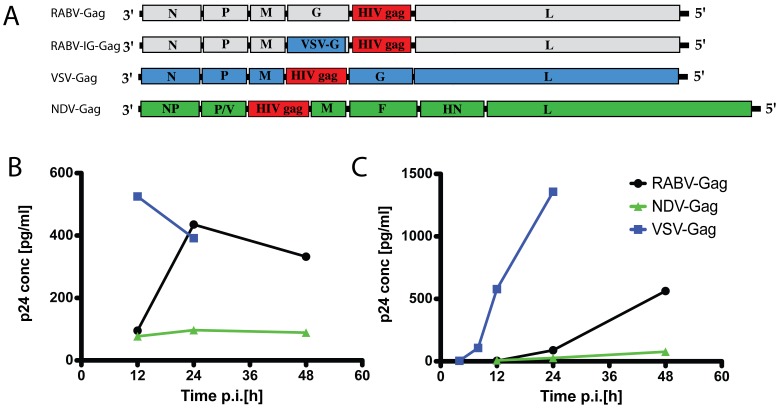
Recombinant vectors and p24 expression levels. (A) The genomes of different recombinant vectors expressing HIV-1 Gag protein used throughout this study are illustrated. The representation of the different genes is not to scale. F, fusion protein; G, glycoprotein; HN, haemagglutinin-neuraminidase protein; L, polymerase; M, matrix protein; N, nucleoprotein; NP, nucleocapsid protein; P, phosphoprotein; P/V, phosphoprotein. (B–C) For quantification of HIV-1 Gag expression levels by p24 ELISA, VERO cells were infected at a MOI of 5 with RABV-Gag, VSV-Gag, and NDV-Gag. Cell lysates (B) and supernatant (C) were collected at the times indicated and p24 antigen levels were measured by ELISA.

The vaccine vectors (Figure 1A) in this study have been previously characterized. RABV-Gag has been previously published as BNSP-Gag [15]. We constructed a RABV-vaccine vector expressing optimized HIV-1 Gag (Optgag) using cDNA published [18], but characterization of this vector revealed lower levels of expression of Gag in the cells and supernatants of infected cells compared to RABV-Gag (data not shown). SPBN-Ig-Gag is the RABV vector in which the RABV glycoprotein gene was exchanged with the ectodomain and transmembrane domain of VSV Indiana strain fused to the cytoplasmic domain of RABV-G [41], [42]. In this paper it is cited as RABV-Ig-Gag.

VSV-Gag was generated from the VSV-M51R, which is attenuated due to the mutation in the matrix protein [29]–[31]. The HIV-1 Gag in the VSV-Gag vector is codon optimized Gag. The codon-optimized Gag gene [18] was amplified by PCR using the primers 5′GGGGTTAATTAAGCCACCATGGGCGTGAG-3′ and 5′-GGGGTTAATTAACTACTGGTCTC-3′. The PCR product was cleaved with Pac I and cloned into the Pac I site of the M protein mutant VSV B5R cDNA infectious clone described in [31]. Infectious virus was recovered from plasmid DNA, and virus stocks were prepared as described [43]. The correct orientation and sequence of the insert was confirmed by sequencing the plasmid DNA and cDNA prepared by RT-PCR of the genome of the recovered virus. Characterization of this vaccine construct by western blot analysis showed increased expression levels of Gag in cells infected with the vector and in the supernatant (data not shown). As such this was used in our studies.

The NDV vector expressing codon-optimized Gag gene has been previously characterized and published [18] and is designated as NDV-Gag in this paper.

### Quantification of HIV-1 Gag– p24 ELISA

VERO cells were seeded in a 12-well plate (2×10^5^ cells per well) the day before infection. The following day they were infected at a multiplicity of infection (MOI) of five with BNSP-gag, VSV-optGag, and NDV-optGag in 250 µl serum-free Optimem medium. After 1 h at 37°C the inoculum was removed and replaced with 1 ml fresh Optimem medium and incubation was continued at 37°C. Supernatant was collected at the times indicated in the figure, spun at 10.000 rpm for 3 min and transferred to a fresh tube. Cells were washed once with 1 ml PBS and then resuspended in lysis buffer [40 mM HEPES pH 7.5, 120 mM NaCl, 1% Triton-X100, 0.4% NaDOC, 1 mM EDTA] supplemented with 1% HALT protease inhibitor cocktail (ThermoScientific Inc.). Lysate was centrifuged for 10 min at 14.000 rpm and transferred to fresh tubes. Protein concentration in the lysate was measured with a BCA kit (Thermofisher) and adjusted to 0.1 µg/µl by the addition of lysis buffer. Supernatant and lysate were diluted with Optimem and PBS, respectively, and assayed for p24 with a RETROTEK HIV-1 p24 Antigen ELISA kit according to the manufacturers instructions (ZeptroMetrix, Buffalo NY).

### Immunization and Challenge Protocol

Mice were primed intramuscularly (i.m.) with either 1×10^6^ foci forming units (ffu) of RABV-Gag (Day 0), 1×10^6^ ffu VSV-Gag (Day 0), or 5×10^6^ ffu NDV-Gag (on Day 0 and 6) in 100 µL administered as two injections –50 µL per hind limb.

For the boost experiments, the mice were rested for 35 days after the prime and then boosted i.m. with either 1×10^6^ ffu RABV-IG-Gag, 1×10^6^ ffu VSV-Gag, 5×10^6^ ffu NDV-Gag (on Day 29 and 35), or did not receive a boost as a control (n = 5 per group). PBS only mice were included as a negative control. Mice were euthanized 5 days after boost, and spleens were harvested for analysis of the cellular response.

For the challenge experiments, the mice were rested for 33 days after the boost and then challenged intraperitoneally (i.p.) with 1×10^6^ plaque forming units (pfu) of recombinant vaccinia virus expressing HIV-1 Gag (vP1287, ARRRP – cited as VV-Gag) in 300 µL (n = 5 per group). Mice receiving PBS only were included as a negative control. Mice were euthanized 5 days after challenge, and spleens were harvested for analysis of the recall response.

### FACS Analysis

Cells were stained for flow cytometry as previously described [44]. In brief, the spleen from the mouse was homogenized and a single-cell suspension of splenocytes obtained. The cells were blocked for 1 hour at 4°C in 100 µl FACS buffer (2% Bovine Serum Albumin in PBS) containing 2 µl Fc block (anti-CD16/32, BD Biosciences) and 3.3 µl unconjugated streptavidin per 1×10^6^ cells in order to reduce non-specific binding by the streptavidin-conjugated tetramer antibody. The cells were washed twice in FACS buffer and then stained for 30 minutes at room temperature with fluorochrome-linked antibodies against cell surface antigens: PerCP-CD8α (BD Biosciences), APC-CD62L (BD Biosciences), and PE-AMQMLKETI tetramer (Becton Dickinson). After surface staining, cells were washed and fixed with 4% paraformaldehyde (BD-cytofix buffer, BD Biosciences) for 20 minutes at 4°C. Cells were washed and re-suspended in FACS buffer and analyzed on an LSRII machine (BD) at the Kimmel Cancer Center Cytometry Facility (Thomas Jefferson University); 200,000 events were collected from each immunized mouse. When analyzing flow cytometry data from splenocytes, the cells were first gated on viability (using forward and side scatter) and then gated for CD8α. All FACS data were analyzed using FlowJo (version 9.5.3) software.

### Intracellular Staining

For the functional analysis of T cells, 1×10^6^ splenocytes/ml were plated in 96-well plate in the presence of GolgiPlug (BD Biosciences) and GolgiStop (BD Biosciences) followed by an incubation for 6 hours at 34°C and 5% CO_2_. For each mouse, two groups were analyzed; the antigen-stimulated group pulsed with 10ug/mL AMQMLKETI peptide and the non-stimulated group left without peptide. The cells were incubated with fluorochrome-linked antibodies against surface proteins - PerCP-CD8α (BD Biosciences) - for 30 minutes at room temperature. Cells were washed and fixed with 4% paraformaldehyde (BD-cytofix buffer, BD Biosciences) for 20 minutes at 4°C, after which cells were washed in Perm/Wash Buffer (BD Bioscience). Cells were then stained for 30 minutes at room temperature in Perm/Wash Buffer containing antibodies against internal antigens: APC-TNFα (BD Pharmingen) and PE-IFNγ (Becton Dickinson). Cells were re-suspended in FACS buffer and FACS analysis was performed as described above.

### Statistical Analysis

All data were analyzed by Prism software (GraphPad, version 5.0d). Statistical analysis was performed using unpaired *t*-test to compare two groups and represented as two-tailed p-value with a confidence interval of 95%. One-way ANOVA with Bonferroni post-test analysis was used where more than two groups were compared. Presented results show the mean of measurements within a group. For all statistics, the following notations are used to indicate significance between two groups: *p<0.05, **p<0.01, ***p<0.001, ****p<0.0001.

## Results

### Utilized Vaccine Vectors Expressing HIV-1 Gag

The vaccine vectors for this heterologous prime/boost approach were RABV-Gag, RABV-IG-Gag (which expresses VSV-Indiana G instead of RABV G), VSV-Gag, or NDV-Gag (Figure 1A). To analyze Gag expression levels for the three vaccine vectors, VERO cells were infected at a MOI of 5 with BNSP-Gag, VSV-Gag, and NDV-Gag. Cell lysates (Figure 1B) and supernatants (Figure 1C) were then collected and at the times indicated p24 antigen was measured by ELISA. Our results indicate that VSV-Gag did express the highest amount of HIV-1 Gag whereas NDV expressed the lowest level of Gag as detected by p24 ELISA. Surprisingly RABV expressed more p24 than detected for NDV-Gag even though the RABV-based vector does not contain the codon-optimized *Gag* utilized for the other two vectors.

### Boost with NDV-Gag in Mice Primed with RABV-Gag and VSV-Gag

**Figure 2:**
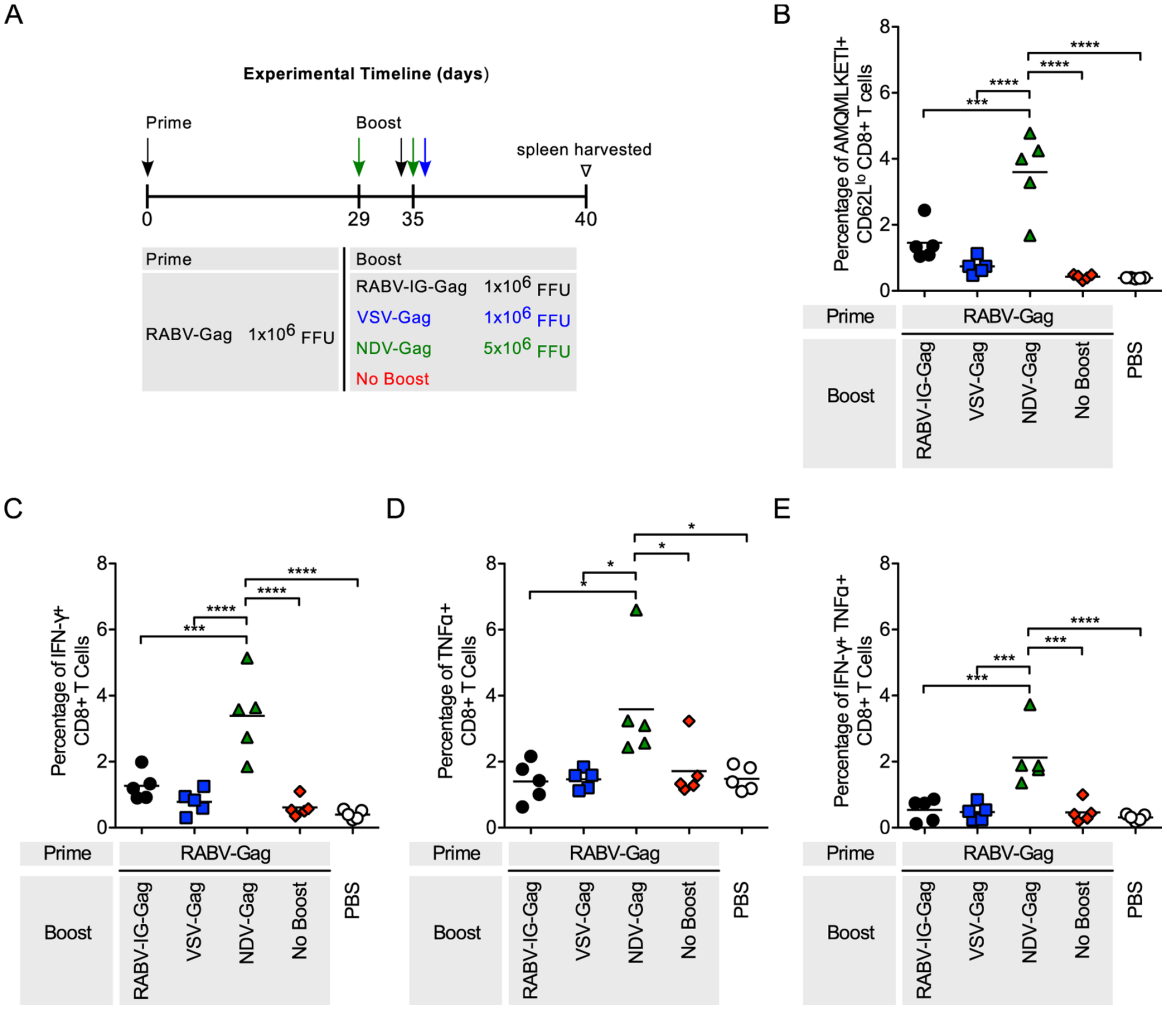
Analysis of Gag-specific CD8^+^ T Cells in the spleen after RABV-Gag prime and heterologous boost. (A) Schedule of prime-boost vaccinations. Mice were primed intramuscularly. Mice mock-immunized with PBS were included as a negative control. (B–D) Each point is representative of splenocytes from one mouse (n = 5 per group). (B) The quantity of activated HIV-1 Gag-specific CD8^+^ T cells in the spleen was analyzed by flow cytometry and the percentage of cells are shown. Activated cells were determined by gating on CD62L^lo^ cells and HIV-1 Gag-specific cells were determined by tetramer staining against the H2^d^ restricted AMQMKLETI epitope. (C–E) The functionality of the CD8^+^ T cells was measured by intracellular cytokine staining for (C) IFNγ^+^, (D) TNFα^+^, and (E) IFNγ^+^TNFα^+^ cells after stimulation of the cells with AMQMKLETI peptide. Statistical analysis was performed using One-WayANOVA. Results shown are presented as the mean. *p<0.05, **p<0.01, ***p<0.001, ****p<0.0001.

CD8^+^ T cells are important effector cells in the control of HIV along with the humoral response against this virus, which is not studied here [45], [46]. Induction of an effective T cell response requires replication of the vectors to achieve adequate antigen expression levels in both the priming and boosting phases. As such heterologous vaccine regimens might facilitate sufficient replication of the vectors to enhance the resulting cellular immune response, since they avoid the problem of anti-vector immunity in the boosting phase that is typical of homologous prime/boost protocols. To analyze the benefits of a heterologous prime/boost approach, we immunized mice with RABV-Gag followed by a second immunization with RABV-IG-Gag (which expresses VSV-Indiana G instead of RABV G), VSV-Gag, or NDV-Gag (Figure 2A). Of note, throughout this study NDV-Gag was always applied twice 6 days apart, because the initial NDV infection does not prevent a second immunization by vector-specific antibodies as observed for RABV and VSV [13]. Two additional groups of mice did not receive any boost or were given an equal volume of PBS as a control. Following the boost immunization, the percentage of HIV-1 Gag specific cells was measured by AMQMLKETI tetramer on activated CD62L^lo^ CD8^+^ T cells (Figure 2B). Functional expression of cytokines by CD8^+^ T cells was analyzed by intracellular cytokine staining (Figure 2C–E). As shown in Figure 2, mice immunized with NDV-Gag had significantly higher induction of Gag-specific CD8^+^ cells compared to VSV-Gag (p<0.0001) and RABV-IG-Gag (p<0.01). Similarly, a higher percentage of activated CD8^+^ T cells from mice boosted with NDV-Gag expressed IFN-γ (p<0.0001), TNF-α (p<0.05), or both IFN-γ and TNF-α (p<0.001).

**Figure 3:**
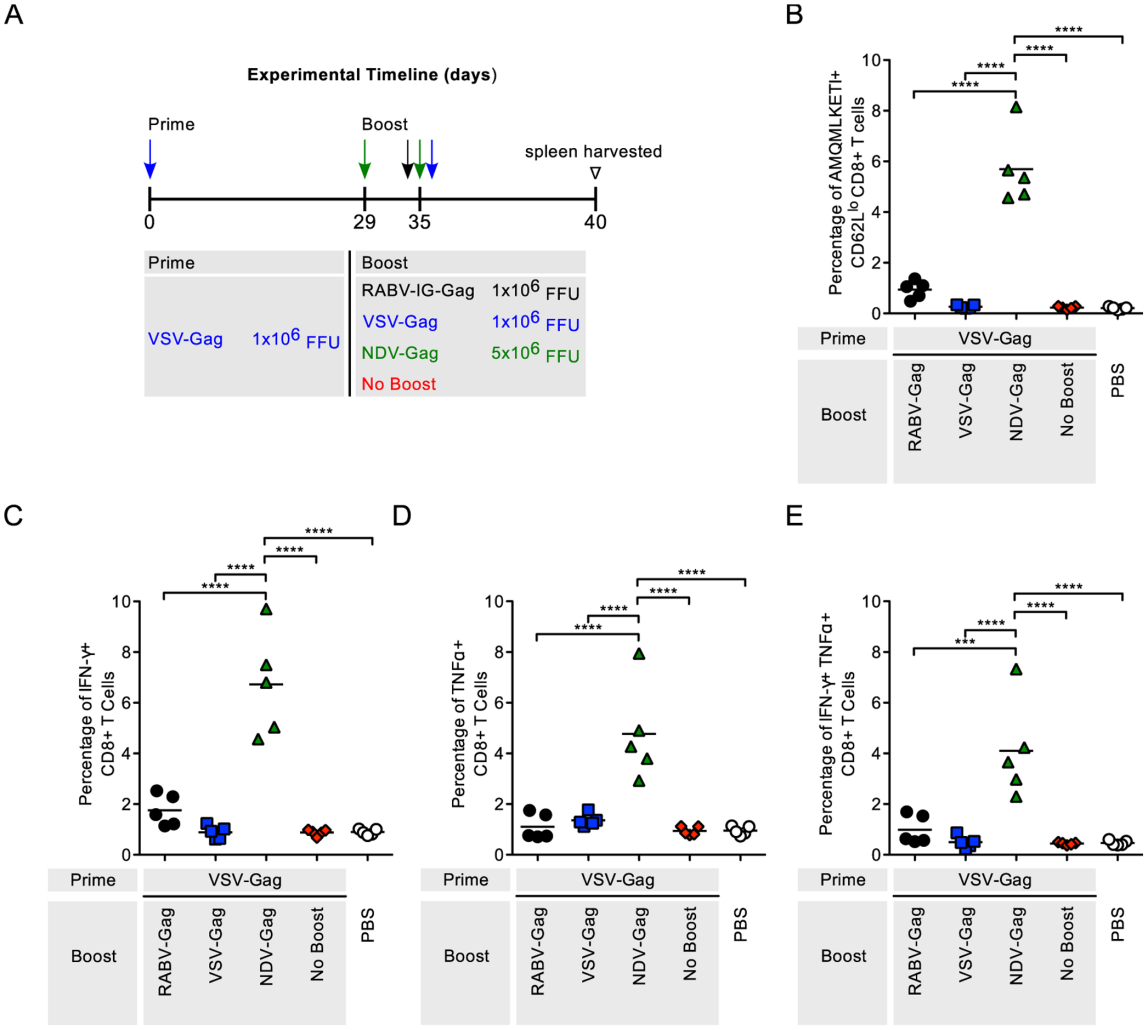
Analysis of the Gag-specific CD8^+^ T Cells in the spleen after VSV-Gag prime and heterologous boost. (A) Schedule of prime-boost vaccinations. Mice were primed intramuscularly. 35 days later the mice were boosted. Mice mock-immunized with PBS were included as a negative control. 5 days after boost, mice were euthanized and spleens harvested for analysis of the cellular response. (B–D) Each point is representative of splenocytes from one mouse (n = 5 per group). (B) The quantity of activated HIV-1 Gag-specific CD8^+^ T cells in the spleen was analyzed by flow cytometry and the percentage of cells are shown. Activated cells were determined by gating on CD62L^lo^ cells and HIV-1 Gag-specific cells were determined by tetramer staining against the H2^d^ restricted AMQMKLETI epitope. (C–E) The functionality of the CD8^+^ T cells was measured by intracellular cytokine staining for (C) IFNγ^+^, (D) TNFα^+^, and (E) IFNγ^+^TNFα^+^ cells after stimulation of the cells with AMQMKLETI peptide. Statistical analysis was performed using One-WayANOVA. Results shown are presented as the mean. *p<0.05, **p<0.01, ***p<0.001, ****p<0.0001.

Similar trends were seen in mice primed with VSV-Gag followed by boosting with RABV-Gag or NDV-Gag (Figure 3A). NDV-Gag induced more robust CD8^+^ T cell responses compared to all other groups (p<0.001) (Figure 3B). Expression of inflammatory cytokines was similar to the level of activation (IFN-γ (p<0.0001), TNF-α (p<0.0001), IFN-γ and TNF-α (p<0.001–0.0001)) (Figure 3C).

### CD8^+^ T Cell Responses in Mice Primed with NDV-Gag

**Figure 4:**
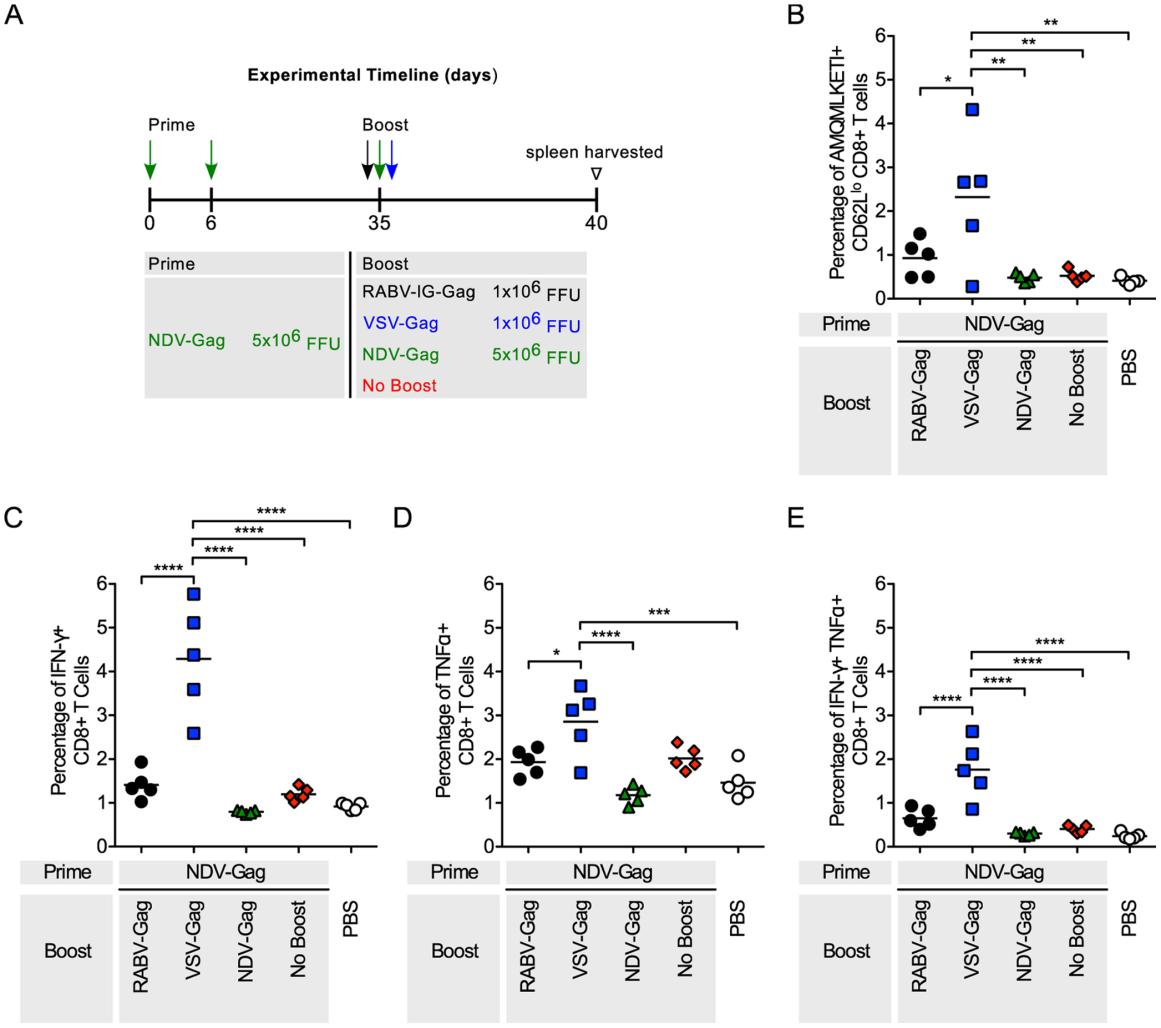
Analysis of the Gag-specific CD8^+^ T Cells in the spleen after NDV-Gag prime and heterologous boost. (A) Schedule of prime-boost vaccinations. Mice were primed intramuscularly on Day 0 and 6. 35 days later the mice were boosted. Mice mock-immunized with PBS were included as a negative control. 5 days after boost, mice were euthanized and spleens harvested for analysis of the cellular response. (B–D) Each point is representative of splenocytes from one mouse (n = 5 per group). (B) The quantity of activated HIV-1 Gag-specific CD8^+^ T cells in the spleen was analyzed by flow cytometry and the percentage of cells are shown. Activated cells were determined by gating on CD62L^lo^ cells and HIV-1 Gag-specific cells were determined by tetramer staining against the H2^d^ restricted AMQMKLETI epitope. (C–E) The functionality of the CD8^+^ T cells was measured by intracellular cytokine staining for (C) IFNγ^+^, (D) TNFα^+^, and (E) IFNγ^+^TNFα^+^ cells after stimulation of the cells with AMQMKLETI peptide. Statistical analysis was performed using One-WayANOVA. Results shown are presented as the mean. *p<0.05, **p<0.01, ***p<0.001, ****p<0.0001.

Mice were primed with NDV-Gag followed by heterologous immunizations with VSV-Gag or RABV-Gag as shown (Figure 4A). Both regimens increased the CD8^+^ T cell response to NDV-Gag compared to the controls (Figure 4B). VSV-Gag induced significantly more HIV-1 Gag specific CD8^+^ T cells than RABV-Gag (p<0.05). A similar trend was seen with expression of IFN-γ (p<0.0001), TNF- α (p<0.05), and IFN-γTNF-α (p<0.0001) (Figure 4C–E). Not surprisingly, homologous responses with NDV-Gag were lower than RABV-Gag or VSV-Gag. Of note, IFN-γ and TNF-α expression in mice that were primed and boosted with NDV-Gag were lower than mice that were not boosted (Figure 4C, D). Taken together, VSV-Gag effectively increased the CD8^+^ T cell response after NDV-Gag prime, whereas this increase was not seen at a similar level after the boost with the RABV-based vaccine vector.

### CD8^+^ T Cell Responses in Vaccine Groups after Challenge with a Recombinant Vaccinia Virus Expressing HIV-1 Gag

**Figure 5:**
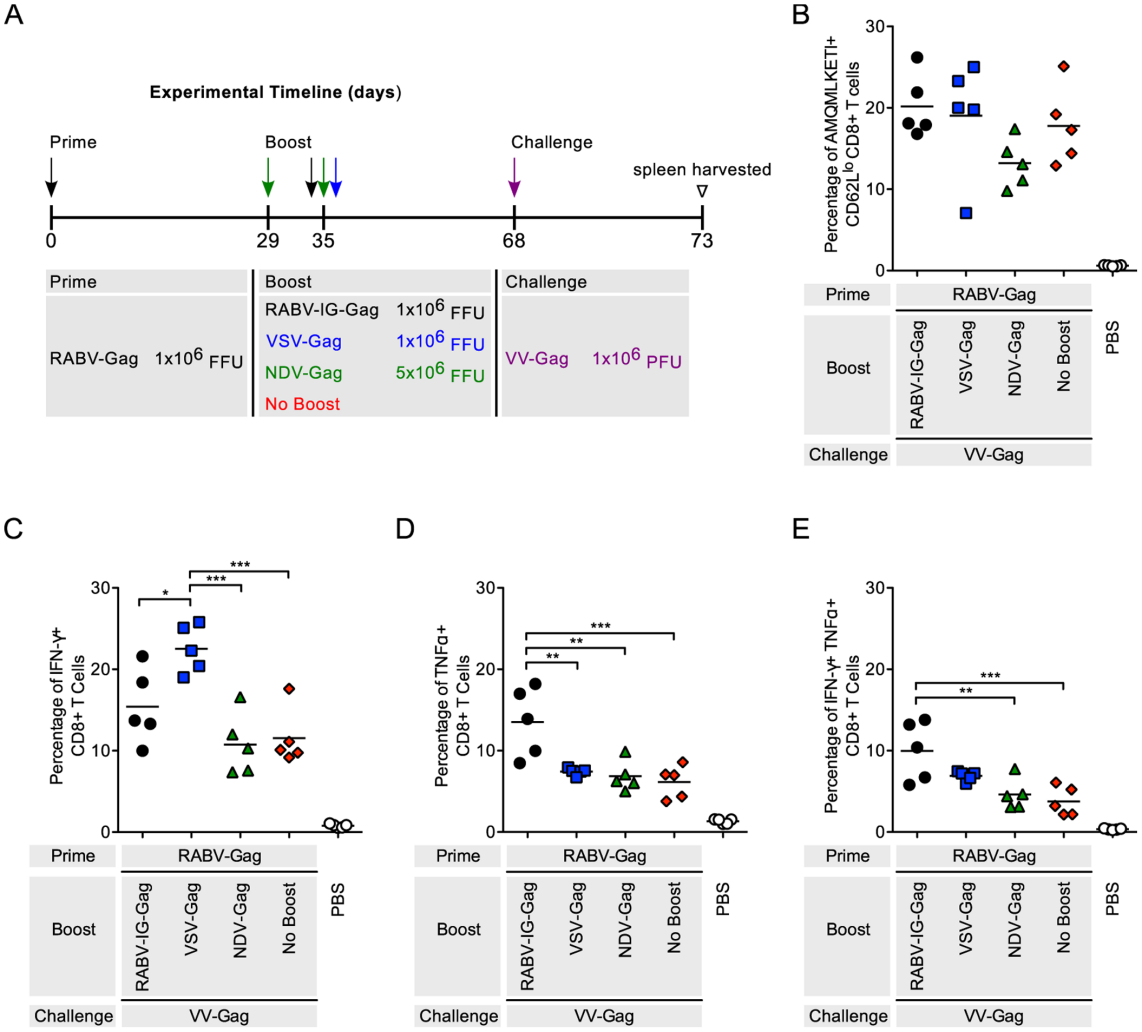
Recall response in mice after RABV-Gag prime and heterologous boost. (A) Schedule of prime-boost-challenge vaccinations. Mice were primed intramuscularly. 35 days later the mice were boosted. Mice were challenged intraperitoneally 33 days post prime. PBS mice were included as a negative control. 5 days after challenge, mice were euthanized and spleens harvested for analysis of the cellular response. (B–D) Each point is representative of splenocytes from one mouse (n = 5 per group). (B) The quantity of activated HIV-1 Gag-specific CD8^+^ T cells in the spleen was analyzed by flow cytometry and the percentage of cells are shown. Activated cells were determined by gating on CD62L^lo^ cells and HIV-1 Gag-specific cells were determined by tetramer staining against the H2^d^ restricted AMQMKLETI epitope. (C–E) The functionality of the CD8^+^ T cells was measured by intracellular cytokine staining for (C) IFNγ^+^, (D) TNFα^+^, and (E) IFNγ^+^TNFα^+^ cells after stimulation of the cells with AMQMKLETI peptide. Statistical analysis was performed using One-Way ANOVA. All comparisons to PBS are statistically significant. Results shown are presented as the mean. **p<0.05, **p<0.01, ***p<0.001, ****p<0.0001.

**Figure 6:**
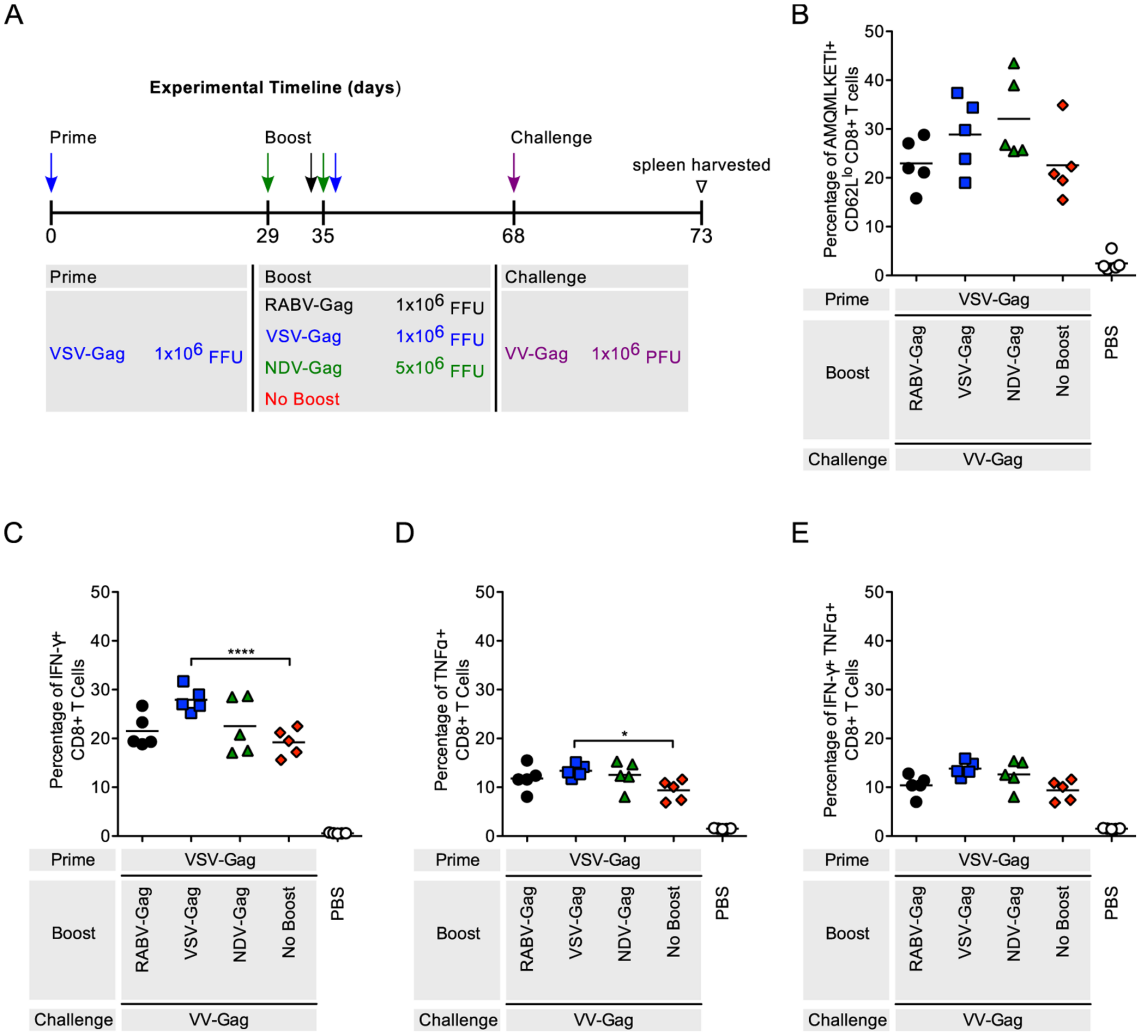
Recall response in mice after a VSV-Gag prime and heterologous boost. (A) Schedule of prime-boost-challenge vaccinations. Mice were primed intramuscularly. 35 days later the mice were boosted. Mice were challenged intraperitoneally 33 days post prime. PBS mice were included as a negative control. 5 days after challenge, mice were euthanized and spleens harvested for analysis of the cellular response. (B–D) Each point is representative of splenocytes from one mouse (n = 5 per group). (B) The quantity of activated HIV-1 Gag-specific CD8^+^ T cells in the spleen was analyzed by flow cytometry and the percentage of cells are shown. Activated cells were determined by gating on CD62L^lo^ cells and HIV-1 Gag-specific cells were determined by tetramer staining against the H2^d^ restricted AMQMKLETI epitope. (C–E) The functionality of the CD8^+^ T cells was measured by intracellular cytokine staining for (C) IFNγ^+^, (D) TNFα^+^, and (E) IFNγ^+^TNFα^+^ cells after stimulation of the cells with AMQMKLETI peptide. Statistical analysis was performed using one-WAY ANOVA. All comparisons to PBS are statistically significant. Results shown are presented as the mean. *p<0.05, **p<0.01, ***p<0.001, ****p<0.0001.

While the boost responses the boost responses after immunization with a heterologous vaccine vector might predict the efficiency of the approach, the final goal of a vaccine is the induction of long-term memory cells, which will control future infection through a recall response. Therefore, mice primed with RABV-Gag followed by heterologous immunizations with the other vectors were challenged with VV-Gag as shown (Figure 5A). The percentage of HIV-1 Gag CD8^+^ T cells (Figure 5B), as well as the expression of IFN-γ and TNF-α, was analyzed as above (Figure 5C–E). Of note, although NDV-Gag induced the most robust CD8^+^ T cell response when used to boost mice immunized with the other NSVs, mice that had been boosted with NDV-Gag had a lower recall response after VV-Gag challenge. Differences between NDV-Gag and VSV-Gag in IFN-γ^+^ cells were statistically significant (p<0.001). In the second series of challenge experiments, mice that were primed with VSV-Gag followed by booster immunization with the other NSVs were challenged with VV-Gag (Figure 6). In general, challenge with VV-Gag resulted in strong responses in all groups compared to the PBS only group, which did not induce any HIV-1 Gag-specific cells. Subtle differences in percentage of IFN-γ^+^ (p<0.0001) and TNF-α^+^ (p<0.05) cells were evident when comparing boosted group responses to those from animals that received VSV-Gag prime only.

**Figure 7:**
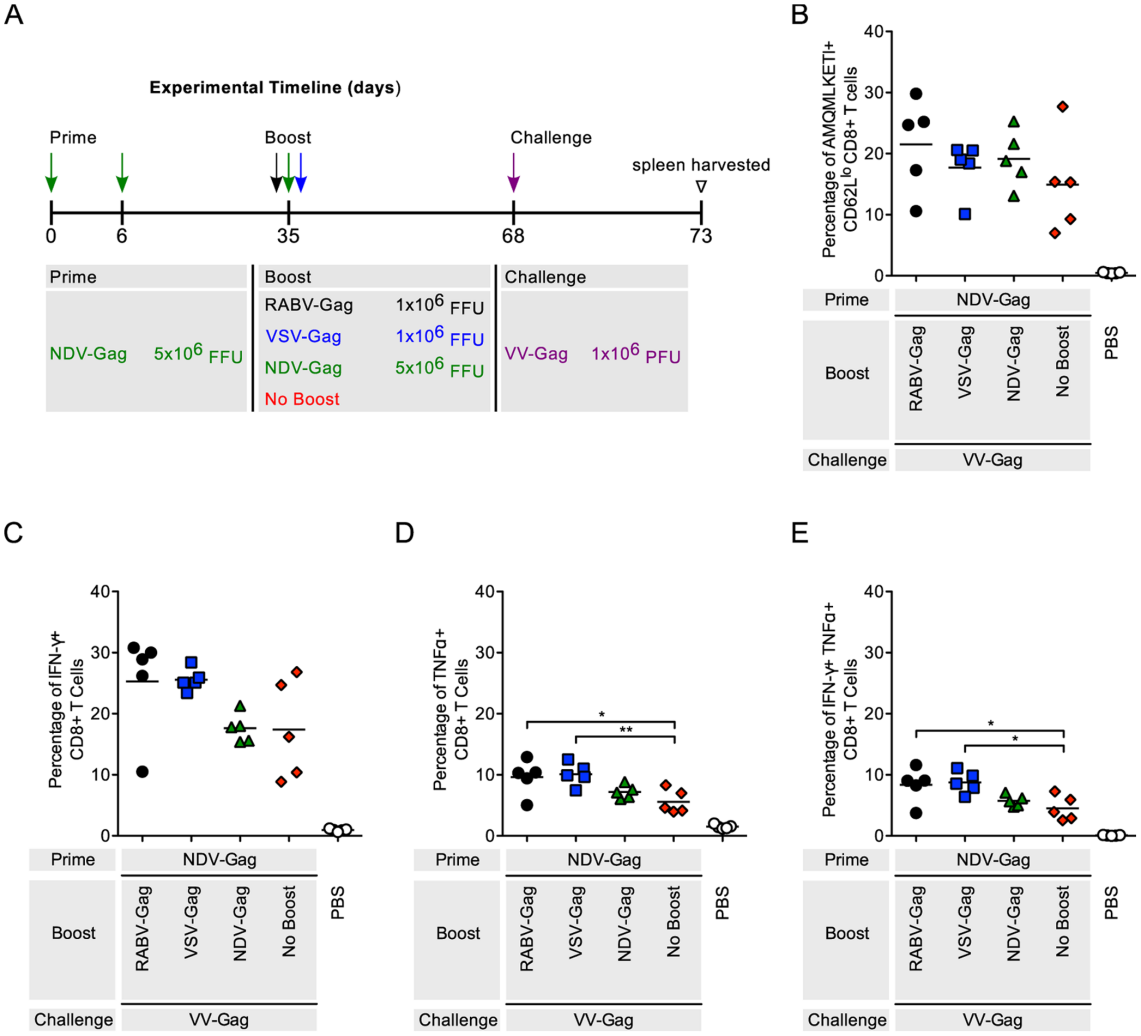
Recall response in mice after NDV-Gag prime and heterologous boost. (A) Schedule of prime-boost-challenge vaccinations. Mice were primed intramuscularly on Day 0 and 6. 35 days later the mice were boosted. Mice were challenged intraperitoneally 33 days post prime. PBS mice were included as a negative control. 5 days after challenge, mice were euthanized and spleens harvested for analysis of the cellular response. (B–D) Each point is representative of splenocytes from one mouse (n = 5 per group). (B) The quantity of activated HIV-1 Gag-specific CD8^+^ T cells in the spleen was analyzed by flow cytometry and the percentage of cells are shown. Activated cells were determined by gating on CD62L^lo^ cells and HIV-1 Gag-specific cells were determined by tetramer staining against the H2^d^ restricted AMQMKLETI epitope. (C–E) The functionality of the CD8^+^ T cells was measured by intracellular cytokine staining for (C) IFNγ^+^, (D) TNFα^+^, and (E) IFNγ^+^TNFα^+^ cells after stimulation of the cells with AMQMKLETI peptide. Statistical analysis was performed using one-WAY ANOVA. All comparisons to PBS are statistically significant. Results shown are presented as the mean. *p<0.05, **p<0.01, ***p<0.001, ****p<0.0001.

The third experimental group of NDV-primed animals were heterologously boosted and then challenged with VV-Gag as described (Figure 7A). Animals boosted with RABV-Gag or VSV-Gag generated significantly higher percentages of TNF- α^+^cells (p<0.05) compared to mice that were not boosted. Similarly, VSV-Gag induced higher expression of TNF-α than unboosted mice (p<0.01). Cells that co-express IFN-γ and TNF-α were higher in both RABV-Gag and VSV-Gag boosted animals compared to unboosted (p<0.05).

### Comparison of Challenge Responses Across All Groups

A comparison of the post-challenge cellular immune responses of all the groups included in this study was performed to identify combinations that induced robust immune responses. Overall VSV-Gag prime induced HIV-1 Gag specific immune responses that were higher than responses in mice primed with RABV-Gag and NDV-Gag (Figure S1A–D). Within the VSV-Gag prime group, boosting twice with NDV-Gag induced the highest amounts of Gag-specific CD8^+^ T cells.

## Discussion

Like many infectious diseases that have had significant impact on the human population, a vaccine against HIV is critically needed. Along with humoral immunity, CD8^+^ T cells have been shown to be important in the control of HIV [45], [46]. Cellular immunity induced in the Ad5-STEP trial failed to protect individuals from HIV, resulting in more people from the vaccine group acquiring infection compared to the placebo [47]. Part of the problem was thought to be the presence of pre-existing immunity against the vector [47], [48]. The NSVs used in this study were chosen, because few individuals have pre-existing immunity.

RABV, VSV and NDV as vaccine vectors against HIV-1 Gag have been extensively studied [15]–[18]. Their genomes are easy to manipulate for insertion of multiple genes without affecting replication [11]–[15]. Furthermore, their pathogenicity and immunogenicity have extensively been studied in mice and NHP [13], [17], [18], [20], [21], [24], [26], [30], [31], [38], [40], and appropriately attenuated vectors have been developed. In this study, we analyzed HIV-1 Gag CD8^+^ T cell responses using each combination of the NSV vectors. Heterologous combinations of the vectors were able to boost HIV-1 Gag CD8^+^ T cells compared to not boosted control animals (Figures 2, 3, 4). As expected, homologous combinations were not as effective at improving the response due to pre-existing immunity to the vector for RABV and VSV. NDV-Gag induced significantly higher CD8^+^ target cell responses in mice primed with RABV-Gag (Figure 2) and VSV-Gag (Figure 3); however, we need to consider that, in the case of NDV-Gag, two boost immunizations were applied. As in previous studies, RABV-IG-Gag was also able to boost responses to RABV-Gag, because it contains the ectodomain and transmembrane domain of the VSV-G Indiana strain [26]. On the other hand, VSV-Gag induced the higher CD8^+^ T cell response in mice primed with NDV-Gag. Together, these post-boost data imply that both NDV-Gag and VSV-Gag are good candidates for use as boost vaccines with any of the three NSVs analyzed in this study. One interesting result of this study was that NDV-Gag was able to efficiently boost Gag-specific CD8^+^ cells after RABV-Gag priming, but VSV-Gag failed to do so (Figure 2B). This finding might help to further elucidate the mechanism of secondary responses for HIV-1 vaccines, and we do suspect that differences in the pattern of cytokine induction pattern might be responsible for the observed effects [26].

It is important to note that the codon-optimized Gag gene inserts were cloned into the VSV and NDV recombinant vectors. Studies with NDV expressing codon-optimized Gag have been published [49]. Optimized Gag expression did not alter NDV recovery, which grew to similar levels as NDV control vectors. Furthermore, increased Gag expression enhanced the CD8^+^ T cell response as determined by reduction in VV-Gag titers in challenge experiments. Similarly, higher levels of HIV-1 Gag were expressed by VSV with optimized HIV-1 Gag than by VSV with non-optimized HIV-1 Gag (data not shown). RABV expressing codon optimized Gag on the other hand did not express higher levels of Gag in the cell lysate or supernatant. In fact, the expression levels of Gag and RABV-N were lower when using codon-optimized Gag compared to the wild-type vector. One possible explanation is that codon optimization leads to differences in viral replication and transcription in the case of RABV. However, Gag expression levels cannot explain the robust post boost responses by VSV-Gag and NDV-Gag (Figure 1B and C), because NDV did express the least amount of HIV Gag while VSV expressed the highest amount of Gag. Of note, both NDV and VSV are potent inducers of type I IFN responses, which might support effective boosting.

The challenge studies in the mice were done using VV-Gag as previously published [50], [51]. VV-Gag is a virus that replicates and induces robust responses against foreign antigens like HIV-1 Gag in mice. The results from the challenge experiment showed that all prime-boost regimens generated significant recall responses compared to naïve controls (Figures 5, 6, 7). Post VV-Gag challenge, mice that had been boosted with NDV-Gag had a lower CD8^+^ T cell response as compared to RABV-Gag and VSV-Gag boosted mice. This is not what we would have expected based on the robust responses induced by NDV-Gag boost data (Figure 2–3). One difference between the boost and challenge studies was the immunization schedule: 35 days between prime and boost compared to 33 days between prime and challenge. However, previous studies have shown good challenge responses at 3 weeks post boost with NDV-Gag [18]. This could potentially be due to differences in long-term memory CD8^+^ T cells.

The functional profile of the cells induced in the immune responses was determined by analyzing expression of IFN-γ and TNF-α. Other cytokines analyzed included IL-2, IL-6 and IL-10. However, the levels of expression of these latter cytokines compared to background were too low and therefore not included (data not shown). In general, expression and co-expression of IFN-γ and TNF-α was proportional to the magnitude of the immune response induced post boost (Figure 2, 3, 4) and post challenge (Figure 5, 6, 7 and Figure S1).

The potential of these vectors as vaccine against HIV-1 Gag has previously been established [15]–[18], [52].

In this study, we show that RABV, VSV and NDV are compatible in a heterologous prime-boost regimen. They are all able to enhance immune responses primed by each other, and they all induced long-term memory cells that could be recalled during challenge. NDV-Gag and VSV-Gag elicited the strongest post boost immune responses likely due to induction of type I interferon responses while VSV-Gag primed mice in general appeared to have the highest responses post challenge. Now that we have established their compatibility, a vaccine regimen with RABV-Gag prime, NDV-Gag Boost #1 and VSV-Gag Boost #2 would be a great candidate to study in mice and macaques with more emphasis on the function of the CD8^+^ T cells. The further addition of envelope proteins for stimulation of humoral responses can be explored.

## Supporting Information

Figure S1 **Comparison of the recall response after the same boost vaccine.** Mice that had been previously immunized and boosted as indicated were challenged intraperitoneally with 1×10^6^ PFU of VV-Gag at 33 days post prime. Five days after challenge, mice were euthanized, and spleens were harvested for analysis of the recall response. (A–D) Each point is representative of splenocytes from one mouse (n = 5 per group). (A) The quantity of activated HIV-1 Gag-specific CD8^+^ T cells in the spleen was analyzed by flow cytometry and the percentage of cells are shown. Activated cells were determined by gating on CD62L^lo^ cells and HIV-1 Gag-specific cells were determined by tetramer staining against the H2^d^ restricted AMQMKLETI epitope. (B–D) The functionality of the CD8^+^ T cells was measured by intracellular cytokine staining for (B) IFNγ^+^, (C) TNFα^+^, and (D) IFNγ^+^TNFα^+^ cells after stimulation of the cells with AMQMKLETI peptide. Statistical analysis was performed using unpaired *t*-test to compare two groups. Results shown are presented as the mean. *p<0.05, **p<0.01, ***p<0.001, ****p<0.0001.
(TIFF)
