# Supplementary figures and images for: Comparison of Heterologous Prime-Boost Strategies against Human Immunodeficiency Virus Type 1 Gag Using Negative Stranded RNA Viruses
Source: PLoS One. 2013 Jun 26;8(6):e67123. doi: 10.1371/journal.pone.0067123 (PMC3694142; doi:10.1371/journal.pone.0067123)

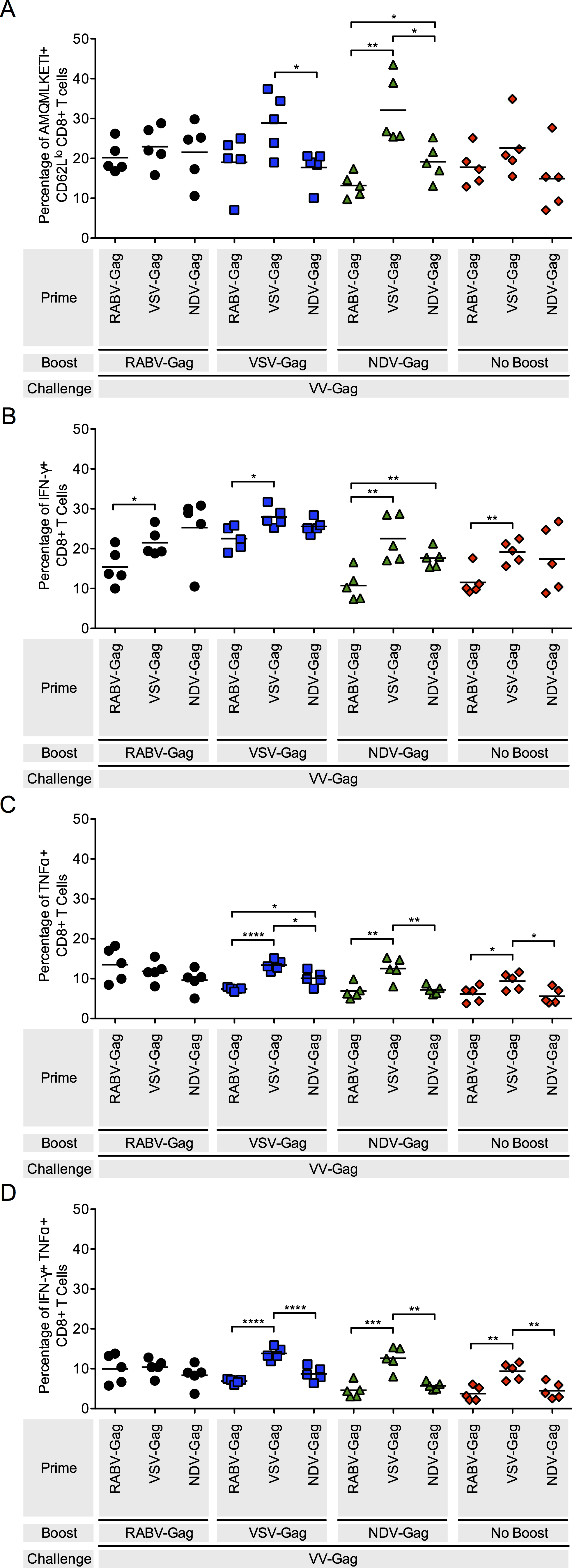

Supplement: Figure S1 — Comparison of the recall response after the same boost vaccine. Mice that had been previously immunized and boosted as indicated were challenged intraperitoneally with 1×10^6^ PFU of VV-Gag at 33 days post prime. Five days after challenge, mice were euthanized, and spleens were harvested for analysis of the recall response. (A–D) Each point is representative of splenocytes from one mouse (n = 5 per group). (A) The quantity of activated HIV-1 Gag-specific CD8+ T cells in the spleen was analyzed by flow cytometry and the percentage of cells are shown. Activated cells were determined by gating on CD62Llo cells and HIV-1 Gag-specific cells were determined by tetramer staining against the H2d restricted AMQMKLETI epitope. (B–D) The functionality of the CD8+ T cells was measured by intracellular cytokine staining for (B) IFNγ+, (C) TNFα+, and (D) IFNγ+TNFα+ cells after stimulation of the cells with AMQMKLETI peptide. Statistical analysis was performed using unpaired t-test to compare two groups. Results shown are presented as the mean. *p<0.05, **p<0.01, ***p<0.001, ****p<0.0001. (TIFF) [file pone.0067123.s001.tiff]
